# The Improvement in the Clinical Management of Patients Undergoing Palliative Care After Training the Primary Care Team by a Specialized Team: A Systematic Review

**DOI:** 10.3390/medsci14060690

**Published:** 2026-10-21

**Authors:** Aila Davis Fanstone Pina Vieira, Deise Aparecida de A. P. Oliveira, Tamyris M. F. Sudré, Lucas Azevedo N. de Carvalho, Gabriela E. Nascimento, Keyci S. Ito Tabata, Rodrigo F. de Oliveira, Vera Lúcia dos S. Alves

**Affiliations:** 1 Graduate Program in Health Sciences, Faculty of Medical Sciences of Santa Casa de São Paulo (PPGCS), São Paulo 01224-001, SP, Brazil; ailadavis@gmail.com (A.D.F.P.V.); keyciflavio@gmail.com (K.S.I.T.); vera.alves@santacasasp.org.br (V.L.d.S.A.); 2 Human Movement and Rehabilitation Graduate Program, Evangelical University of Goiás (UniEVANGÉLICA), Anápolis 75083-515, GO, Brazil; drlucasnog@gmail.com (L.A.N.d.C.); gabievangelista.n@gmail.com (G.E.N.); rodrigofranco65@gmail.com (R.F.d.O.); 3 Undergraduate Program in Physiotherapy, Evangelical University of Goiás (UniEVANGÉLICA), Anápolis 75083-515, GO, Brazil; tamyrismendesf@hotmail.com

**Keywords:** palliative care, primary care, coaching, signs and symptoms

## Abstract

**Background:** Primary Health Care plays an important role in the provision and coordination of palliative care in many healthcare contexts. Education and training of healthcare professionals are relevant strategies to support the delivery of quality palliative care. **Objective:** To evaluate whether training healthcare professionals working in Primary Health Care improves symptom assessment and clinical management of patients eligible for palliative care. **Methodology:** Systematic review conducted according to PRISMA 2020 guidelines, with a protocol registered on the PROSPERO platform under the number CRD420261370621. The eligibility criteria were structured according to the PICO approach: population composed of healthcare professionals working in primary care; educational interventions in palliative care; comparator without training or with different modalities; outcomes of symptom assessment and clinical control. The searches were conducted in seven databases: PubMed/MEDLINE, Embase, Web of Science, Scopus, Cochrane Library, SciELO, and LILACS. **Results:** Eight studies were included in the review, with varied designs: a pilot randomized clinical trial, a quasi-experimental study, two evaluations of educational programs, a feasibility study, a retrospective population cohort, and two qualitative approaches. The findings revealed a generalist predominance in the provision of palliative care: 95% of the care was provided by generalists, 53% of patients did not receive identifiable palliative care in the last year of life, and specialists were virtually nonexistent in rural areas, where family doctors were responsible for nearly all care. **Conclusions:** Training of primary care teams is necessary, but insufficient on its own. Although it improves the confidence and knowledge of professionals, there is no robust evidence that it sustainably transforms patient clinical outcomes. The problem is structural: there is a lack of adequate clinical time, compatible remuneration, integration with specialists, and support infrastructure. It is necessary to go beyond education and promote reforms in the healthcare system so that palliative care in primary care is effectively qualified.

## 1. Introduction

Primary Health Care (PHC) constitutes the structural axis of contemporary health systems, serving as the main entry point and coordinator of longitudinal care, with a recognized impact on reducing health inequalities and the sustainability of systems [1,2]. In the current context of accelerated demographic transition and increasing prevalence of multimorbidities, primary care teams assume a central role in managing chronic conditions, requiring robust clinical competencies for systematic symptom assessment and effective therapeutic control [3,4].

In this scenario, educational interventions aimed at training primary care teams, encompassing continuing education programs, simulation-based training, peer mentoring, and the development of specific competencies, emerge as priority strategies to qualify the care process [5,6]. Primary studies suggest that such strategies can contribute to the standardization of clinical practices, increased adherence to evidence-based protocols, and improved early recognition of warning signs, although the effects show considerable variability across different contexts and populations [7,8].

The integration of specialized teams and APS professionals, mediated by structured training programs, fosters the exchange of technical-scientific knowledge and strengthens evidence-based practice [9]. Integrated care models that incorporate team training demonstrate potential to improve continuity of care and care coordination, although robust evidence on objective clinical outcomes is still limited [8,10].

The qualification of APS teams directly influences the ability to stratify risk, implement timely interventions, and coordinate longitudinal care, critical elements for the effective management of patients with long-term health conditions [5,7]. However, the specific impact of team training on symptom assessment and clinical control remains poorly systematized, with primary studies showing methodological heterogeneity, variability in the outcomes evaluated, and gaps regarding contextual characteristics that enhance the effects of interventions [6].

Primary Health Care (PHC) constitutes the privileged setting for the provision of basic palliative care, given its reach and longitudinal bond with patients and families throughout the course of the disease. Unlike the hospital-school model, primary care allows for the integration of palliative principles, relief of suffering, communication about care goals, and advance planning in the daily routine of basic health units and households [11]. Studies show that the incorporation of palliative care in primary health care reduces unnecessary hospitalizations, improves symptom control, and strengthens patients’ autonomy in the context of their social integration [12,13]. However, the effectiveness of this integration directly depends on the training of the teams, which often report insecurity in addressing complex symptoms and identifying patients eligible for palliative care [14].

Given the dispersion of evidence and the absence of recent systematic reviews on the effects of training interventions for Primary Health Care (PHC) teams, it becomes imperative to rigorously investigate the impact of these strategies on symptom assessment and clinical control of patients. The present systematic review aims to fill this gap by evaluating the effectiveness of training conducted by specialized teams in the qualification of the care provided, focusing on the improvement of systematic symptom assessment and clinical control of eligible patients. It also seeks to analyze to what extent this training contributes to the standardization of care practices, optimization of clinical decision-making, and improvement of health outcomes, providing robust support for evidence-based recommendations and guiding policies for ongoing health education.

## 2. Methods

The present study is configured as a systematic literature review, conducted according to the methodological guidelines recommended by the Preferred Reporting Items for Systematic Reviews and Meta-Analyses (PRISMA 2020) [15], with the purpose of synthesizing and critically evaluating the available scientific evidence on the impact of training Primary Health Care (PHC) teams on symptom assessment and clinical control of patients (Supplementary Materials PRISMA 2020 Checklist).

Before the start of the systematic searches, the protocol for this review was prospectively registered on the International Prospective Register of Systematic Reviews (PROSPERO) platform, under the identification number CRD420261370621.

### 2.1. Formulation of the Research Question and PICO Structuring

The guiding question of this systematic review was structured according to the PICO framework [16]. The central question that guided this investigation was “What is the impact of training healthcare professionals working in Primary Health Care on symptom assessment and clinical management of patients eligible for palliative care?” The eligibility criteria are presented in Table 1.

The PICO framework was selected because the primary objective of this systematic review was to investigate the impact of an educational intervention on predefined clinical outcomes. This framework allows the population, intervention, comparator, and outcomes to be explicitly defined, thereby providing a structure that is consistent with the interventional nature of the research question. Although the SPC framework may be useful for exploratory or qualitative questions focused on a specific situation, perspective, or context, it was considered less appropriate for the present review because the study specifically sought to examine the effects of training interventions on symptom assessment and clinical management. Therefore, PICO was considered the most suitable framework for operationalizing the eligibility criteria and guiding the literature search.

The population of interest (P) included healthcare professionals from different professional categories working in Primary Health Care, whether evaluated individually or as members of multiprofessional primary healthcare teams. These included family physicians, general practitioners, nurses, nursing technicians, community health workers, pharmacists, physiotherapists, nutritionists, psychologists, and dentists, as well as professionals working in Basic Health Units, Health Centers, health posts, or equivalent services. The intervention of interest (I) consisted of educational strategies aimed at developing clinical competencies for symptom assessment and management of chronic conditions. The comparator group (C) included professionals or teams without structured educational intervention, as well as subgroups exposed to different modalities or intensities of training. The outcomes evaluated (O) were stratified into two main categories: outcomes related to symptom assessment, including diagnostic capacity and early recognition; secondary outcomes included advance care planning, communication about goals of care, referral or integration with specialized palliative care services, interprofessional collaboration, and implementation-related outcomes, when reported by the included studies. For the purposes of this review, professional capacity was considered in relation to outcomes such as knowledge, confidence, competence, clinical management capacity, interprofessional collaboration, and self-reported changes in clinical practice, when assessed by the included studies. The term capacity building was not interpreted as evidence of sustained organizational or health-system capacity.

### 2.2. Search Strategy and Information Sources

The search strategy was developed collaboratively by members of the review team, with expertise in PHC, continuing health education, and research methodology, aiming to ensure adequate sensitivity and specificity in retrieving relevant studies. A comprehensive literature search was conducted on 10 March 2026, across PubMed/MEDLINE, Embase, Web of Science Core Collection, Scopus, Cochrane Library, SciELO, and LILACS.

The construction of the search strategies was based on the use of controlled descriptors from the DeCS (Health Sciences Descriptors) and MeSH (Medical Subject Headings) structures, complemented by free terms and synonyms relevant to the investigated topic. The terms were organized into five conceptual domains: palliative care, primary health care, professional training, symptom management and clinical outcomes, and study designs. The combinations between the domains were carried out using Boolean operators (“OR” for terms within the same domain and “AND” for intersection between domains), excluding systematic reviews, editorials, and protocols. No publication year restrictions were applied during the initial searches, and studies were not excluded based on publication date. Therefore, no predefined time limit was used for study identification. The complete search strategies, detailed for each database, are presented in Table 2. A systematic search of gray literature was not performed. However, reference lists of included studies were manually screened, and thesis repositories and conference proceedings were consulted as to identify potentially relevant studies.

Studies were excluded when the primary outcomes of interest were assessed exclusively using non-validated or psychometrically unsupported instruments.

Measurement instruments: studies were eligible when the outcomes of interest were assessed using validated or psychometrically established instruments appropriate to the population and outcome domain. No specific instrument was required a priori, provided that the instrument had evidence of validity or established psychometric properties for the construct being assessed. Non-validated instruments, when used as the sole measure of an outcome of interest, were excluded.

### 2.3. Study Selection and Screening Process

All retrieved records were imported into Rayyan© (1.7.8) [17] for reference organization, duplicate identification, and study screening. The selection process was conducted independently and in duplicate by previously trained reviewers. Two independent reviewers screened the titles and abstracts of all retrieved records, followed by full-text assessment of potentially eligible reports by two other independent reviewers, according to the predefined inclusion and exclusion criteria. Disagreements were recorded and resolved by consensus; when consensus was not reached, a third reviewer performed the final assessment.

At all stages, the evaluations were conducted in a blinded manner regarding the decisions of the other reviewer in the pair, in order to minimize agreement bias. Any disagreements between the reviewers were systematically recorded on the platform and resolved through consensus discussion; when consensus was not reached, they were resolved by a third evaluation with greater methodological expertise, ensuring greater rigor and reliability before proceeding to final arbitration. In addition to the database searches, the reference lists of systematic reviews and meta-analyses identified during the search process were screened to identify potentially relevant primary studies that may not have been retrieved through the electronic database searches. Potentially eligible primary studies identified through this process were subjected to the same eligibility criteria and screening procedures as records retrieved from the databases.

**Figure 1:**
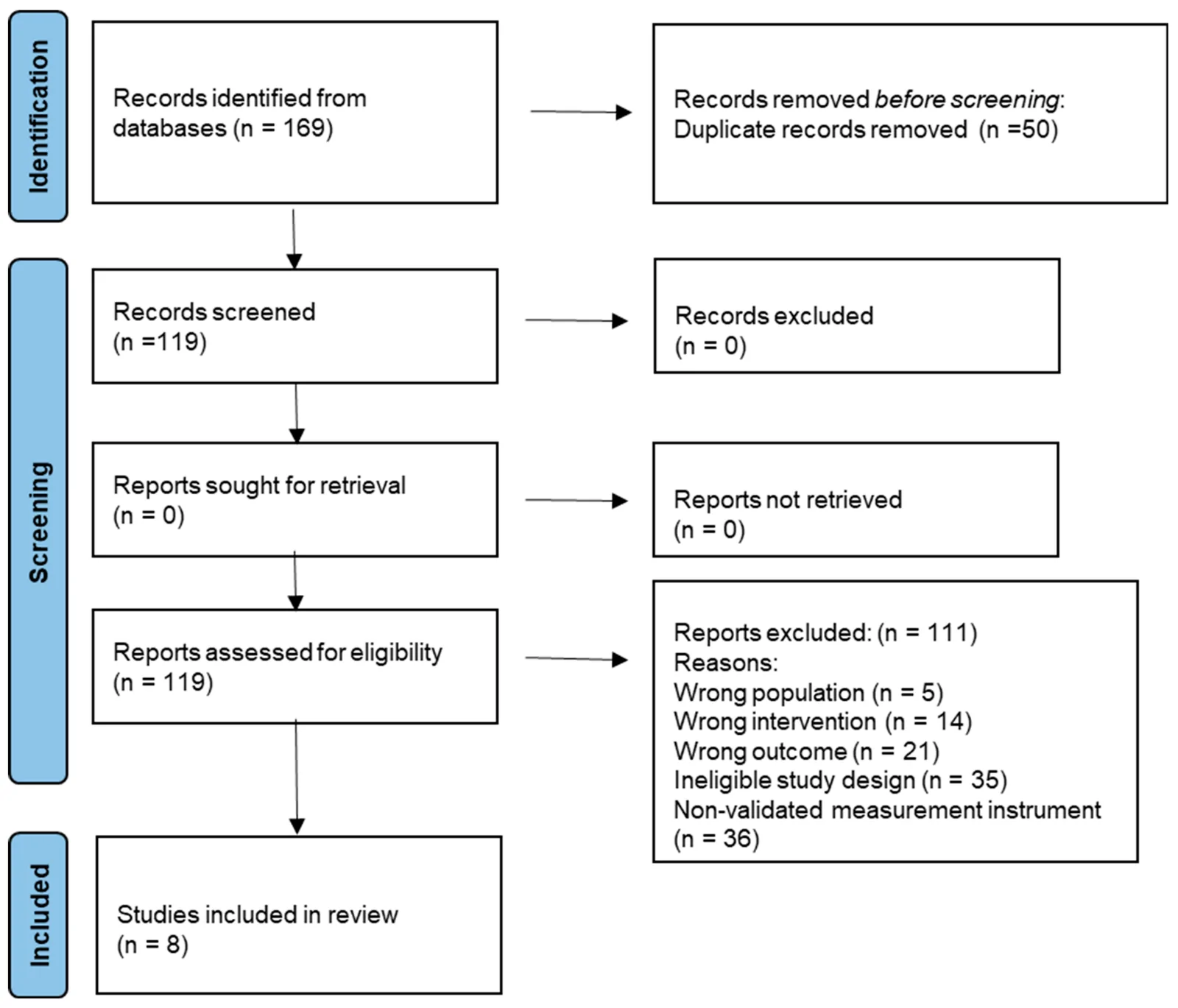
PRISMA, 2020. Source: Adapted by the authors, 2026.

At the full-text assessment stage, each excluded report was assigned a predefined primary reason for exclusion. The specific reasons and corresponding numbers of excluded reports are presented in the PRISMA 2020 flow diagram [15] (Figure 1).

### 2.4. Complementary Eligibility Criteria

Studies were eligible when they provided data relevant to the assessment of the predefined outcomes and allowed the characterization of the effects, experiences, or clinical implications of the educational intervention. Both quantitative and qualitative approaches were considered eligible, provided that they addressed the research question and met the predefined population, intervention, comparator, and outcome criteria.

Studies were excluded when they did not provide data relevant to the predefined outcomes, did not address the research question, or did not meet the predefined population, intervention, comparator, or methodological eligibility criteria.

Abstracts from conferences, editorials, letters to the editor, comments, narrative reviews without systematic analysis, case reports or case series with a sample size of fewer than 30 participants, duplicate studies or studies with data published redundantly in multiple articles (keeping only the most complete or recent publication), studies with insufficient data for extraction even after contacting the authors, and studies that only assess perceptions, satisfaction, or attitudes of professionals without measuring clinical outcomes in patients were excluded.

### 2.5. Data Extraction

The data extraction was conducted in a standardized and independent manner by two reviewers, using an electronic form specifically developed for this review. The instrument was previously tested on a pilot sample of five studies and refined as necessary. The extracted variables were organized into seven main categories: bibliographic characteristics, including authors, year of publication, country of origin, language of the article, source of funding, and declared conflicts of interest; methodological characteristics, including study design, location of implementation, recruitment period, sample calculation, and ethical considerations; population characteristics, encompassing sample size, response rate, losses and follow-up, included professional categories (doctors, nurses, technicians, community agents, and others), time of practice in Primary Health Care, and service characteristics (urban, rural, and size of the assigned population); intervention characteristics, including type of training (continuing education, simulation, mentoring, clinical supervision, and update course), duration, frequency, modality (in-person, remote, and hybrid), topics covered, professionals responsible for training, and existence of protocols or guidelines used; outcome characteristics, including symptom assessment instruments employed (with specification of names and psychometric properties), clinical control indicators measured (with specification of physiological parameters), evaluation moments (pre, post, and follow-up), and description of main results; statistical analyses, including the effect measures used (mean difference, odds ratio, risk ratio, hazard ratio, and Cohen’s d), 95% confidence intervals, *p*-values, adjustments for confounding variables, and subgroup or interaction analyses performed; and, finally, adverse events or barriers to implementation reported, when applicable.

The extractions performed by each reviewer were compared, and discrepancies were resolved by consensus or arbitration by a third reviewer. When essential data were missing or ambiguous, the authors of the original studies were contacted by email, up to two attempts, requesting clarifications or additional data.

### 2.6. Evaluation of Study Quality and Risk of Bias

This systematic review was conducted according to the guidelines of the Preferred Reporting Items for Systematic Reviews and Meta-Analyses (PRISMA 2020) [15]. The search strategy was developed in electronic databases (PubMed/MEDLINE, Web of Science, Scopus, SciElo, and LILACS) using controlled descriptors (MeSH/DeCS) and keywords combined with Boolean operators, without language or publication period restrictions. Inter-rater agreement was not quantified using Cohen’s kappa in the original review process; the reviewers independently screened the records, and disagreements were resolved by consensus or by a third reviewer.

The complete flow of the selection process, including the number of records identified, duplicates removed, screened, assessed in full, excluded with justification, and finally included, is detailed in the PRISMA [15] flowchart. At the full-text assessment stage, each excluded report was assigned a predefined primary reason for exclusion. The specific reasons and corresponding numbers of excluded reports are presented in the PRISMA 2020 flow diagram (Figure 1).

Data extraction was carried out in a standardized and independent manner by two reviewers, using a previously tested electronic form. The characteristics of the included studies, encompassing author, year, country, design, participants (inclusion criteria, sample size, demographic characteristics), interventions and comparators, primary and secondary outcomes, were systematized and are presented in Table 1.

The assessment of the risk of bias in the included randomized clinical trials was conducted using the Risk of Bias 2 (RoB 2) tool [18], the revised version of 2019, which examines five domains: (1) randomization process, (2) deviations from the intended intervention protocol, (3) missing outcome data, (4) outcome measurement, and (5) selection of the reported outcome. Each domain was classified as low risk of bias, some concerns, or high risk of bias, resulting in an overall judgment per study. The results of this assessment are presented in Table 2, and the graphical representation of the bias domains can be found in Table 2.

The synthesis of the results was structured according to the GRADE framework (Grading of Recommendations Assessment, Development and Evaluation) [19] to assess the certainty of the evidence by outcome. The classification started at high certainty for randomized clinical trials, and was subsequently downgraded or maintained based on five factors: risk of bias, inconsistency, indirectness, imprecision, and publication bias. The final certainty was categorized as high, moderate, low, or very low. The GRADE evidence framework (Summary of Findings), which synthesizes the estimated effects, the magnitude of the results, and the confidence in the estimate for each previously defined critical or important outcome, is presented in Table 3.

## 3. Results

For the synthesis of the evidence, a systematic extraction of the methodological data and findings from eight (8) studies included in this review was carried out. The information was organized into standardized categories that allowed for comparison between the investigations regarding the characteristics of the samples, the measurement instruments used, the training interventions for the evaluated primary healthcare teams (theoretical, practical, hybrid, in-person, and distance training), and the outcomes analyzed.

Considering the diversity of study designs, predominantly randomized clinical trials and quasi-experimental studies, with some program evaluation studies and previous systematic reviews, and the heterogeneity of the investigated populations (family doctors, nurses, nursing technicians, community health agents, and multiprofessional teams from basic health units), the presentation of data in tabular format aims to facilitate the identification of association patterns between training interventions and the outcomes of symptom assessment and clinical control of patients eligible for palliative care, as well as the evaluation of the quality and consistency of the available evidence.

Table 4 presents the detailed extraction of the included studies, organized according to the following domains: bibliographic reference, sample characteristics, instruments and tests used, investigated exposure factors, prevalent outcome/pain, and main results found.

### 3.1. Characterization of the Included Studies

The present systematic review synthesized evidence from eight primary studies that investigated the impact of training and capacity building of Primary Health Care teams on the assessment of symptoms and clinical management of patients with palliative care needs. Regarding the methodological designs, one study adopted a pilot randomized clinical trial [25], one used a two-phase quasi-experimental design [27], two evaluated implemented educational programs [21,22], one configured a feasibility study [20], one constituted a retrospective population cohort [26], and two employed qualitative approaches [23,24]. The geographical distribution focused on high-income countries, with the exception of Kwok et al. (2022) [22], who investigated Caribbean countries. Canada [26], the United States by two [26,27], the United Kingdom by two [21,23], and Scotland, Australia, and the Caribbean by one study each [20,22,24].

### 3.2. Provision of Palliative Care in PHC: Predominance of Generalists, Scarcity of Specialists, and Access Inequalities

Brown et al. (2018) [26], in a retrospective population cohort study conducted in Ontario, Canada, between April 2011 and March 2015, analyzed data from 361,951 adult decedents and 11,006 physicians who provided palliative care. The authors identified four models of provision: low engagement (53.0% of the deceased did not receive medical palliative care); exclusive generalist (21.2%); consultation, or shared care between generalists and specialists (14.7%); and exclusive specialist (11.1%). Among the doctors who provided palliative care, 95.3% had a generalist focus and 4.7% had a specialist focus. The distribution by medical specialty indicated that 74.2% were family doctors, 8.3% internists, 3.1% oncologists, and 2.1% general surgeons. Only 12.1% of general practitioners and 2.7% of palliative care specialists worked in rural areas, where more than 99% of palliative care was provided by family doctors.

These findings converge with Reymond et al. (2005) [24], who, in an Australian context, highlighted the urgent need for educational strategies aimed at primary healthcare professionals in rural areas, where physical distance and a shortage of human resources make specialized care practically inaccessible. The comparison between Brown et al. (2018) [26] and Reymond et al. (2005) [24], conducted on different continents and with an interval of more than a decade, reveals a structural constancy: primary health care, especially in rural areas, functions as the only point of access to palliative care, without the professionals who comprise it necessarily having the specialized training for such care. This transnational and temporal convergence suggests that the dependence of PHC for providing palliative care is a systemic phenomenon, not confined to specific health contexts, but intrinsic to the organization of health systems that prioritize primary care as the entry point.

The shortage of palliative care specialists in primary health care (PHC), documented by Brown et al. (2018) [26], is indirectly corroborated by Kimbell et al. (2018) [20], who, in a Scottish context, tested a 6-month intervention led by a specialist nurse in supportive liver care, acting as a bridge between PHC and specialized services. The need for an “embedded” specialist in PHC to enable care coordination and advance planning paradoxically reinforces the gap identified by Brown et al. (2018) [26]; in the absence of specialists, palliative care in PHC becomes, at best, generalist and poorly coordinated, and at worst, nonexistent. The feasibility demonstrated by Kimbell et al. (2018) [20], universal acceptability among patients, caregivers, and professionals, as well as a significant increase in shared electronic care plans between APS and hospitals, indicates that the presence of a specialist, even if temporary, substantially modifies the quality of the care process, but does not resolve the structural issue of scarcity.

### 3.3. Feasibility and Acceptability of Educational Interventions: Diversity of Models and Implementation Contexts

The diversity of educational interventions identified in this review reflects the absence of a single, universally accepted model for training primary care teams in palliative care, as well as the adaptation of strategies to local contexts of resources and infrastructure. Matthew et al. (2023) [21] evaluated the CAPACITI program, implemented in London, United Kingdom, consisting of virtual education with 10 modules lasting 1 h each per month, aimed at 22 primary care teams and 159 participants. The structure combined interactive webinars, quick reference materials, 30-day practical tasks, and mentorship with a palliative care specialist. The thematic analysis of qualitative data from 15 teams in focus groups and 21 teams in reflection surveys identified three themes: changes in practice, usefulness of the components, and barriers and challenges to implementation. The participants reported that the multifaceted approach was useful for building trust and competence, and the main changes in practice were the early identification of patients and the initiation of conversations about serious illness.

Kwok et al. (2022) [22], in a completely different context, Caribbean countries with extremely limited access to specialists and poor technological infrastructure, adopted the Project ECHO model, based on tele-mentoring with regular case discussion sessions and interactive teaching via videoconference. Despite the contextual differences, the United Kingdom with a universal healthcare system and robust infrastructure versus the Caribbean with scarce resources, both studies converged in demonstrating the feasibility and acceptability of distance educational models, with a measurable impact on participants’ confidence and knowledge. Matthew et al. (2023) [21] emphasized the building of trust and competence through a multifaceted approach, while Kwok et al. (2022) [22] highlighted the strengthening of interprofessional collaboration and the scalability of the intervention in contexts with limited access. This transcontextual convergence suggests that distance education, when structured and combined with elements of interactivity and mentoring, overcomes geographical and economic barriers, although both studies have evaluated process outcomes (trust, knowledge, self-reported changes) and not objective clinical outcomes in patients.

The contrast between these virtual models and in-person or hybrid interventions offers additional insights into the contingencies of implementation. Kavalieratos et al. (2022) [25], in the PENPal-HF trial, tested training for outpatient cardiology nurses in palliative care principles, applied in in-person quarterly visits over 56 weeks. Adherence was 70% until week 56, with 93.75% patient satisfaction and 87.5% agreement on the need for intervention expansion. Kimbell et al. (2018) [20], in turn, used a hybrid approach: a nurse specialist in supportive liver care working in person for 6 months, with care coordination, advance planning, and continuous support. The acceptability was universal, with a significant increase in electronic care plans shared between primary care and hospitals.

Reymond et al. 2005 [24], in a rural Australian context, combined training, specialist support, and skill development for the management of advanced diseases, reporting improvements in professionals’ confidence and competence, an increase in the capacity to provide care locally, and the strengthening of integration with specialized services. The comparison between CAPACITI (virtual, self-directed, scalable), Project ECHO (virtual, interactive, case-centered), PENPal-HF (in-person, integrated into clinical routine), Kimbell’s intervention (hybrid, with embedded specialist), and Reymond’s model (in-person, with external support) suggests that there is no single optimal approach, but rather a continuum of strategies whose effectiveness depends on the local context, available resources, and specific barriers to be overcome. However, none of these studies directly compared modalities with each other, making it impossible to infer the relative superiority of each model.

### 3.4. Impact on Symptom Assessment and Quality of Life: Promising but Fragmented Evidence

The central domain of this review, the impact of training APS teams on symptom assessment and clinical control, presented evidence in two studies with experimental or quasi-experimental designs, both with methodological limitations that restrict causal inference.

Kavalieratos et al. (2022) [25], in the only randomized clinical trial included, demonstrated that training outpatient cardiology nurses in palliative care principles resulted in self-reported improvement in 93.75% of the intervention group patients, who reported improvements in physical symptoms, mood, and quality of life. The intervention incorporated systematic assessment of symptom burden through the Edmonton Symptom Assessment Scale-Revised, prioritization of patient preferences, and advance care planning, applied in a structured manner during quarterly visits. However, the study did not present a formal statistical comparison between groups for these outcomes, limiting causal inference. Moreover, the sample was small (*N* = 30), with a high mortality rate (5 deaths during the study), and the intervention was conducted by outpatient cardiology nurses, a relatively structured context that may not reflect the reality of primary health care in less advantaged contexts.

Nguyen et al. (2018) [27], in a quasi-experimental two-phase design, investigated a nurse-led palliative care intervention adapted for community practice in patients with non-small cell lung cancer (stages II to IV) and their family caregivers, recruited from three community centers of the Kaiser Permanente system in California. The results showed a significant improvement in the physical, emotional, and functional domains of quality of life (*p* < 0.01), maintained for up to 3 months. Caregivers showed improvement in physical and spiritual well-being and preparedness for care. Although this study was not strictly conducted in primary health care, the patients were recruited from community centers within an integrated health system; it reinforces the feasibility of transferring palliative care competencies to first-level care professionals, with a measurable impact on multiple domains of quality of life.

The comparison between these findings and the studies that evaluated process outcomes reveals an important gap. Kimbell et al. (2018) [20] validated the Palliative Care Outcome Scale and the EuroQol-5D-5L as appropriate instruments for measuring outcomes in palliative care in primary health care, but did not measure direct clinical outcomes in patients. Forbat et al. (2024) [23] evaluated qualitative outcomes, increased team confidence and competence, improved communication, strengthened integration between services, favoring care in the preferred location, and reduction in potentially avoidable hospitalizations, but without objective measurement of symptoms in patients.

This disconnection between care processes (more discussions, more documented plans, greater team confidence) and patient outcomes (quality of life, symptom burden, place of death) constitutes a critical gap in the current literature.

The assessment instruments used in the studies also deserve consideration. Kimbell et al. (2018) [20] validated the POS and EuroQol-5D-5L as suitable for measuring outcomes in palliative care in PHC. Kavalieratos et al. (2022) [25] successfully used the ESAS-r and the Kansas City Cardiomyopathy Questionnaire, in addition to other psychometric instruments. Nguyen et al. (2018) [27] employed the FACT-L, FACIT-Sp, and CoH-QOL. The diversity of instruments, some specific to palliative care, others generic for quality of life, and some specific to conditions, reflects the complexity of measuring outcomes in palliative care, where the multiplicity of symptoms requires multidimensional approaches. However, this diversity makes it difficult to directly compare studies and accumulate evidence on which instruments are more sensitive to changes produced by specific educational interventions.

### 3.5. Advance Care Planning and Communication: Between Theory and Practice

Advance care planning and discussions about care goals are central components of palliative care, representing the transition from a reactive model to a proactive and patient-centered model. However, the findings of this review reveal a discrepancy between the theoretical recognition of the importance of these practices and their effective implementation in PHC.

Brown et al. (2018) [26] did not directly measure advance care planning or goals-of-care discussions; therefore, the absence of identifiable palliative care contact cannot be interpreted as direct evidence of absent advance care planning.

Matthew et al. (2023) [21], in the evaluation of CAPACITI, reported that the main self-reported practice changes by primary care teams were the early identification of patients with palliative needs and the initiation of conversations about serious illness. The authors did not quantify the frequency or depth of these conversations, nor their impact on clinical outcomes or quality of life, but the identification of these changes as primary by the program itself suggests that the initial barrier, the initiation of the conversation, is significant and can be overcome through structured education.

Kavalieratos et al. (2022) [25], in PENPal-HF, incorporated the Advance Care Planning Engagement Survey as a measurement tool, but did not present specific results in this domain in the published findings, limiting themselves to indicating self-reported overall improvement. The absence of detailed data on advance care planning in a randomized clinical trial, the most robust methodological design of the review, reinforces the gap in evidence on this specific outcome.

The comparison between these findings indicates that, while educational programs like CAPACITI can modify the self-reported practice of primary care teams, increasing early identification and the initiation of conversations, the measurement of the impact of these changes on patient outcomes remains nascent.

None of the included studies quantified whether the increase in discussions about care goals, produced by educational interventions, resulted in decisions more aligned with patients’ preferences, reduction in aggressive treatments at the end of life, or improvement in the quality of dying.

### 3.6. Barriers and Facilitators for Implementation: An Integrated Analysis

The cross-analysis of qualitative and implementation studies revealed a set of recurring barriers, which operate at multiple levels and must be considered in the design of future educational interventions.

At the individual level, Matthew et al. (2023) [21] identified barriers and challenges to implementation as the third analytical theme in the CAPACITI evaluation, although they did not detail the specific barriers mentioned by the participants. The lack of detail limits the identification of strategies for overcoming these barriers, but the very existence of the theme as central in the qualitative analysis indicates that the barriers are perceived by professionals as significant.

At the organizational level, Forbat et al. (2024) [23] did not specify particular barriers in the implementation of the Palliative Care Needs Rounds, focusing instead on the positive outcomes of the intervention. However, the formal structure of the meetings, regular, structured, with the participation of specialists and professionals from long-term care institutions, functions as an implicit organizational facilitator, creating a dedicated space for case discussion that would not exist in the usual routine. Kimbell et al. (2018) [20] demonstrated that the presence of an embedded specialist (supportive liver care nurse) acted as a facilitator, creating a stable point of reference for the APS team, although they did not detail the barriers faced.

At the systemic level, Brown et al. (2018) [26] demonstrated that the structure of financing and remuneration of health services directly influences the provision of palliative care in PHC. In Ontario, the fee-for-service model may discourage the time needed for complex discussions about care goals, which are not as well compensated as technical procedures. This inference, although not explicitly tested by the authors, is supported by the low prevalence of medical palliative care in the general population (47% of the deceased) and the concentration of specialists in urban areas.

The integrated analysis of these barriers allows us to identify a central paradox in the literature: while there is consensus on the need to strengthen palliative care competencies in primary health care, the necessary support structures to sustain these competencies, adequate clinical time, aligned remuneration models, formal integration with specialists, and a favorable organizational culture, remain underdeveloped. Successful educational interventions, such as CAPACITI and Project ECHO, seem to have overcome this paradox, at least partially, through approaches that are not limited to individual training but also incorporate elements of organizational change (practical tasks, mentorship, communities of practice).

## 4. Discussion

### 4.1. Training and Capacity Building of Healthcare Professionals in Primary Health Care

The findings of this systematic review indicate that training and educational interventions can improve aspects of professional capacity among healthcare professionals working in Primary Health Care (PHC). Matthew et al. (2023) [21], through the CAPACITI program, and Kwok et al. (2022) [22], through Project ECHO, reported improvements in confidence, knowledge, and competence following educational interventions. Reymond et al. (2005) [24] also reported positive changes following a training program for healthcare professionals working in rural areas.

Despite these findings, the available evidence should be interpreted with caution. The outcomes related to professional capacity were frequently based on self-reported measures, qualitative assessments, or feasibility studies. Improvements in knowledge, confidence, or perceived competence do not necessarily indicate changes in clinical practice or improvements in patient outcomes.

This distinction is particularly relevant to the interpretation of the present findings. Although educational interventions may improve the preparation of healthcare professionals, the included studies provide limited evidence regarding their effects on patient-level outcomes, such as symptom control, hospitalizations, or place of death. Kavalieratos et al. (2022) [25], through the PENPal-HF intervention, reported improvements in symptoms and quality of life. However, the pilot design, the small sample of 30 participants, the five deaths during the study, and the absence of intergroup statistical analysis for specific clinical outcomes limit the interpretation of these findings. Therefore, the results should be considered promising but not conclusive.

### 4.2. Integration of Palliative Care into Primary Health Care

The included studies also highlight the important role of PHC professionals in the provision of palliative care. Brown et al. (2018) [26] reported that 95.3% of medical palliative care in Ontario was provided by generalists, while 53% of deceased patients had no identifiable palliative care contact during the last year of life. These findings illustrate the extent to which generalist professionals may be involved in palliative care and also indicate that access to specialized services remains an important issue.

Palliative care competencies may be relevant throughout the course of advanced chronic diseases rather than being limited to the final stage of life. Professionals caring for patients with advanced heart failure, chronic kidney disease, cirrhosis, and other progressive conditions may need to recognize palliative care needs and incorporate symptom management and communication about goals of care into routine practice.

The availability of specialized professionals may influence the implementation of this approach. Brown et al. (2018) [26] reported that only 4.7% of doctors had a specialized focus in palliative care, with 2.7% working in rural areas. Educational initiatives such as CAPACITI and Project ECHO may help extend specialist knowledge to PHC professionals. Kwok et al. (2022) [22] demonstrated the feasibility of a virtual educational model in the Caribbean. However, the implementation of similar models may depend on factors such as technological infrastructure, internet access, workforce availability, and the organization of healthcare services.

Forbat et al. (2024) [23] evaluated Palliative Care Needs Rounds, which established regular interaction between specialist palliative care teams and long-term care institutions. The study reported increased professional confidence, improvements in advance care planning, and reductions in hospitalizations. Nevertheless, the qualitative design and the need for regular specialist involvement should be considered when interpreting these findings.

Kimbell et al. (2018) [20] evaluated a model in which a specialist nurse in supportive liver care was embedded in PHC for six months. The study reported high acceptability, increased use of shared care plans, and validation of assessment instruments. Although these findings support the feasibility of integrating specialist support into PHC, the sustainability of the changes after the specialist’s withdrawal remains uncertain.

Nguyen et al. (2018) [27], in a quasi-experimental study, reported significant improvements in physical, emotional, and functional quality-of-life domains, which were maintained for three months. Caregivers also experienced improvements. However, the study was conducted within the Kaiser Permanente healthcare system, which has a highly integrated care structure. The applicability of these findings to healthcare systems with different levels of integration and resources should therefore be considered carefully.

### 4.3. Advance Care Planning and Communication

Advance care planning (ACP) and communication about goals of care are important components of palliative care. However, the included studies provided different levels of evidence regarding these aspects.

Brown et al. (2018) [26] did not directly assess ACP or goals-of-care discussions. Therefore, the absence of identifiable palliative care contact among some patients should not be interpreted as direct evidence that advance care planning did not occur.

Matthew et al. (2023) [21] reported an increase in self-reported conversations about serious illness following the CAPACITI intervention. However, the study did not quantify the frequency or depth of these conversations or evaluate their subsequent effects on clinical decision-making. Thus, an increase in communication does not necessarily indicate that patient preferences were incorporated into subsequent care.

These findings highlight the need for future studies to assess not only whether communication occurs, but also its timing, quality, and relationship with clinical decisions. Standardized measures of ACP and communication may also facilitate comparison between interventions and healthcare settings.

### 4.4. Barriers to Implementation

Information regarding barriers to implementation was limited and varied between the included studies. Matthew et al. (2023) [21] identified barriers as one of the themes in their qualitative analysis, whereas other studies focused primarily on feasibility, professional outcomes, or clinical results.

For this reason, the present review does not allow firm conclusions regarding the frequency or magnitude of specific organizational barriers. Factors such as workload, staff turnover, remuneration, organizational culture, and infrastructure may influence the implementation of palliative care interventions, but these aspects were not systematically evaluated across the included studies.

The findings nevertheless suggest that training may be more effective when combined with appropriate organizational and specialist support. Improvements in professional knowledge and confidence may not be sufficient to change clinical practice when professionals have limited time, restricted access to specialist advice, or inadequate communication and referral pathways.

### 4.5. Measurement of Outcomes and Heterogeneity of Evidence

Considerable heterogeneity was observed in the instruments used to assess outcomes. The included studies used measures such as the Palliative Care Outcome Scale (POS), EuroQol-5D-5L, Edmonton Symptom Assessment System-Revised (ESAS-r), Kansas City Cardiomyopathy Questionnaire (KCCQ), Functional Assessment of Cancer Therapy-Lung (FACT-L), Functional Assessment of Chronic Illness Therapy-Spiritual Well-Being (FACIT-Sp), and City of Hope Quality of Life (CoH-QOL).

This variability makes direct comparison between studies difficult. Although Kimbell et al. (2018) [20] supported the use of the POS and EuroQol-5D-5L in their study, the use of an appropriate instrument does not necessarily demonstrate that it is sensitive to changes resulting from professional training.

The methodological diversity of the included studies also affected the interpretation of the results. The evidence included a pilot randomized study, a quasi-experimental study, qualitative investigations, and observational studies. Consequently, the available evidence provides information on feasibility, professional outcomes, and some patient outcomes, but remains insufficient to establish strong causal relationships between training interventions and clinical outcomes.

### 4.6. Limitations of the Review

This review has several limitations. First, the methodological heterogeneity of the included studies, which ranged from a pilot randomized trial and quasi-experimental study to qualitative and observational designs, limited direct comparison between interventions and precluded quantitative meta-analysis. Second, several studies assessed professional outcomes such as confidence, competence, and perceived changes in practice using self-reported or qualitative measures, which limits the strength of causal inference. Third, the evidence was geographically concentrated in high-income countries, reducing the generalizability of the findings to settings with different healthcare structures and resource availability.

The inclusion of studies published across different periods should also be considered when interpreting the findings. Although no publication-year restriction was applied, some included studies, particularly Reymond et al. (2005) [24], reflect healthcare systems, professional training models, and service configurations that may differ from current practice. Changes in palliative care policies, digital education, healthcare organization, workforce composition, and integration between primary and specialized care may limit the direct applicability of older findings to contemporary settings. Therefore, the results should be interpreted considering the temporal and contextual heterogeneity of the included evidence.

Finally, although the search was conducted in the selected databases, studies published in non-indexed sources or in languages outside the eligibility criteria may not have been identified.

### 4.7. Overall Interpretation

Overall, the findings indicate that training interventions may improve knowledge, confidence, competence, and perceived clinical capacity among healthcare professionals working in PHC. However, the evidence regarding the effects of these interventions on patient-level outcomes remains limited.

The results suggest that education should be considered as one component of a broader strategy for integrating palliative care into PHC. Access to specialist support, appropriate referral pathways, adequate clinical time, and effective communication between healthcare services may also influence the extent to which professionals can apply the competencies acquired through training.

Further studies should use comparative designs, standardized professional and patient-level outcomes, and longer follow-up periods to assess whether improvements associated with training are maintained over time. Research conducted in different healthcare systems is also needed to determine whether educational models developed in highly integrated settings can be effectively adapted to other contexts.

## 5. Conclusions

This systematic review demonstrated that Primary Health Care is the main provider of palliative care in most of the analyzed contexts, with family doctors responsible for the majority of the care and specialists concentrated in urban areas. Training and capacity-building interventions were associated with improvements in knowledge, confidence, competence, and self-reported practice in several included studies. However, the evidence of objective clinical impact on symptom assessment and clinical management of patients remains limited in magnitude and generalizability.

Advance care planning is precariously implemented, with a low prevalence of formal discussions about care goals and late referrals to specialists. Structural barriers—unsustainable workload, misaligned compensation models, staff turnover, and lack of functional integration with specialized services—operate at levels that isolated education cannot reach.

It is concluded that training APS teams is a necessary strategy, but insufficient on its own to ensure the quality of palliative care. An integrated approach is essential, combining ongoing education with systemic reforms that protect clinical time, value complex discussions, and strengthen integration between levels of care. Future investigations should prioritize larger randomized clinical trials, with composite outcomes and cost-effectiveness analysis in diverse contexts.

## Figures and Tables

**Table 1:** Eligibility criteria structured according to the PICO approach.

Element	Inclusion Criteria	Exclusion Criteria
P (Population)	Healthcare professionals working in Primary Health Care, evaluated individually or as members of multiprofessional teams in Basic Health Units, Health Centers, health posts, or equivalents; professionals currently active or in training for PHC practice.	Health professionals exclusively working in secondary or tertiary care; undergraduate students without employment ties in primary health care; management professionals without direct clinical practice; emergency care teams or urgent and emergency services without continuity of care in primary health care.
I (Intervention)	Educational interventions, training programs, professional development, continuing education, mentoring, clinical supervision, simulation-based training, workshops, refresher courses, interventions for developing specific clinical competencies for symptom assessment and palliative care management.	Interventions exclusively for administrative management without a clinical educational component; training focused exclusively on administrative or bureaucratic procedures; capacity-building interventions for specialized or hospital care without interface with primary health care.
C (Comparator)	Professionals or primary healthcare teams without educational intervention (usual control group); different modalities or intensities of training (comparative between educational strategies); teams before and after intervention (before and after studies); different contexts of training implementation	Absence of a defined comparator group; exclusive comparison between different medical specialties without focus on PHC; studies solely descriptive of perceptions about training without analysis of clinical outcomes
O (Outcomes)	Symptom assessment: ability to recognize warning signs, diagnostic accuracy, time to symptom identification, adherence to screening protocols, and systematic evaluation (e.g., validated instruments such as Patient Health Questionnaire-9, Generalized Anxiety Disorder-7, Edmonton Symptom Assessment System) Clinical control: indicators of patients with palliative care needs (blood pressure, blood glucose, hemoglobin A1c, body mass index), treatment adherence, rates of avoidable complications, hospitalizations for conditions sensitive to primary care, specific mortality, health-related quality of life (SF-36, EQ-5D)	Studies that do not evaluate objective clinical outcomes or symptom assessment; outcomes exclusively related to professional satisfaction or self-efficacy perception without measuring patient results; administrative process outcomes without clinical impact (e.g., number of correctly filled forms without association with health outcomes)

Source: Produced by the authors, 2026.

**Table 2:** Descriptors used in databases.

Databases	Descriptors
PubMed	(“Palliative Care”[Mesh] OR “Terminal Care”[Mesh] OR “Hospice Care”[Mesh] OR “palliative care”[Title/Abstract] OR “terminal care”[Title/Abstract] OR “end of life care”[Title/Abstract] OR “end-of-life care”[Title/Abstract] OR “supportive care”[Title/Abstract] OR palliat*[Title/Abstract]) AND (“Primary Health Care”[Mesh] OR “Family Practice”[Mesh] OR “primary health care”[Title/Abstract] OR “primary care”[Title/Abstract] OR “family practice”[Title/Abstract] OR “family medicine”[Title/Abstract] OR “family health strategy”[Title/Abstract] OR “community health services”[Title/Abstract]) AND (“Education, Professional”[Mesh] OR “Inservice Training”[Mesh] OR training[Title/Abstract] OR education[Title/Abstract] OR educat*[Title/Abstract] OR “professional training”[Title/Abstract] OR “health personnel training”[Title/Abstract] OR “health personnel education”[Title/Abstract] OR “staff training”[Title/Abstract] OR “staff education”[Title/Abstract] OR “continuing education”[Title/Abstract] OR “capacity building”[Title/Abstract] OR mentorship[Title/Abstract]) AND (“symptom management”[Title/Abstract] OR “symptom control”[Title/Abstract] OR “symptom relief”[Title/Abstract] OR “symptom burden”[Title/Abstract] OR “clinical outcome*”[Title/Abstract] OR “patient outcome*”[Title/Abstract] OR “treatment outcome*”[Title/Abstract] OR “quality of life”[Title/Abstract] OR “pain management”[Title/Abstract]) AND (“Clinical Trial”[Publication Type] OR “Randomized Controlled Trial”[Publication Type] OR “Observational Study”[Publication Type] OR “Cohort Studies”[Mesh] OR trial[Title/Abstract] OR cohort[Title/Abstract] OR “intervention study”[Title/Abstract] OR “implementation study”[Title/Abstract]) NOT (“systematic review”[Title] OR “scoping review”[Title] OR “narrative review”[Title] OR editorial[Publication Type] OR comment[Publication Type] OR letter[Publication Type] OR guideline[Publication Type] OR protocol[Title])
Web of Science	(“palliative care” OR “terminal care” OR “end of life care” OR “end-of-life care” OR “supportive care” OR “hospice care” OR palliat*) AND (“primary health care” OR “primary care” OR “family practice” OR “family medicine” OR “family health strategy” OR “community health services” OR “general practice” OR “community-based care”) AND (training OR education OR educat* OR “professional training” OR “health personnel training” OR “health personnel education” OR “staff training” OR “staff education” OR “continuing education” OR “continuing professional development” OR “capacity building” OR mentorship OR supervision OR “knowledge translation”) AND (“symptom management” OR “symptom control” OR “symptom relief” OR “symptom burden” OR “clinical outcome*” OR “patient outcome*” OR “treatment outcome*” OR “quality of life” OR “pain management”) AND (trial OR “clinical trial” OR “randomized controlled trial” OR “controlled study” OR cohort OR “observational study” OR “intervention study” OR “implementation study” OR “program evaluation”) NOT (“systematic review” OR “scoping review” OR “narrative review” OR “literature review” OR editorial OR commentary OR “letter to the editor” OR guideline OR protocol)
SciELO	(“cuidados paliativos” OR “palliative care”) AND (“atenção primária” OR “atenção primária à saúde” OR “primary care”) AND (capacitação OR treinamento OR training OR education)
Scopus	TITLE-ABS-KEY (“palliative care” OR “terminal care” OR “end of life care” OR “end-of-life care” OR “supportive care” OR “hospice care” OR palliat*) AND TITLE-ABS-KEY (“primary health care” OR “primary care” OR “family practice” OR “family medicine” OR “family health strategy” OR “community health services” OR “general practice” OR “community-based care”) AND TITLE-ABS-KEY (training OR education OR educat* OR “professional training” OR “health personnel training” OR “health personnel education” OR “staff training” OR “staff education” OR “continuing education” OR “continuing professional development” OR “capacity building” OR mentorship OR supervision OR “knowledge translation”) AND TITLE-ABS-KEY (“symptom management” OR “symptom control” OR “symptom relief” OR “symptom burden” OR “clinical outcome*” OR “patient outcome*” OR “treatment outcome*” OR “quality of life” OR “pain management”) AND TITLE-ABS-KEY (trial OR “clinical trial” OR “randomized controlled trial” OR “controlled study” OR cohort OR “observational study” OR “intervention study” OR “implementation study” OR “program evaluation”) AND NOT TITLE-ABS-KEY (“systematic review” OR “scoping review” OR “narrative review” OR “literature review” OR editorial OR commentary OR “letter to the editor” OR guideline OR protocol)

Source: Produced by the authors, 2026.

**Table 3:** GRADE scale.

**Outcomes**	**№ of Participants** **(Studies)** **Follow-Up**	**Certainty of the Evidence** **(GRADE)**	**Relative Effect** **(95% CI)**	**Anticipated Absolute Effects**	
				Risk with Cuidado Usual (Sem Intervenção Educacional Estruturada)	Risk Difference with Treinamento/Capacitação das Equipes de APS em Cuidados Paliativos
Quality of life, assessed with ESAS-r, KCCQ, FACT-L, EuroQol-5D-5L, and POS	(8 non-randomized studies)	⨁◯◯◯ Very low	-	not pooled	not pooled
Symptom control, assessed with ESAS-r, POS, and clinical assessment	(8 non-randomized studies)	⨁◯◯◯ Very low	-	not pooled	not pooled
Hospitalizations, assessed with administrative data and electronic records	(3 non-randomized studies)	⨁◯◯◯ Very low	not pooled	not pooled	not pooled
Team confidence, assessed with self-reported surveys, focus groups, and thematic analysis	(5 non-randomized studies)	⨁◯◯◯ Very low	Five studies [20,21,22,23,24] reported an increase in confidence and competence after training, with a consistent direction of effect. All data were self-reported, without a control group or standardized measurement.		
Advance care planning, assessed with ACP Engagement Survey, electronic care plans, and self-report	(4 non-randomized studies)	⨁◯◯◯ Very low	Four studies [21,22,23,25] documented an increase in care plans and the initiation of conversations about serious illness. Predominantly qualitative data, without quantification of frequency or impact on patient outcomes.		
**The risk in the intervention group (and its 95% confidence interval) is based on the assumed risk in the comparison group and the relative effect of the intervention (and its 95% CI).** **CI: confidence interval**					
**GRADE Working Group grades of evidence** **High certainty: we are very confident that the true effect lies close to that of the estimate of the effect.** **Moderate certainty: we are moderately confident in the effect estimate: the true effect is likely to be close to the estimate of the effect, but there is a possibility that it is substantially different.** **Low certainty: our confidence in the effect estimate is limited: the true effect may be substantially different from the estimate of the effect.** **Very low certainty: we have very little confidence in the effect estimate: the true effect is likely to be substantially different from the estimate of effect.**					

**Table 4:** Data extraction.

Reference	Sample	Instruments/Tests	Exposure/Intervention	Main Outcomes	Results	Risk of Bias Scale (Rob 2)
Kimbell et al., 2018 (Scotland) [20]	*N* = 47 patients with advanced liver disease; 27 family caregivers; 13 professionals involved in the case	Palliative Care Outcome Scale (POS); EuroQol-5D-5L; medical record analysis; semi-structured interviews	6-month intervention conducted by a nurse specialist in supportive liver care: care coordination, advance care planning, support for patients/caregivers, acting as a resource for community professionals	Feasibility and acceptability of the intervention; care coordination; care planning processes; quality of life; resource use	Intervention acceptable to all participants; significant increase in the number of electronic care plans shared between primary care and hospitals; POS and EuroQol-5D-5L identified as appropriate instruments for outcome measurement	High risk of bias
Matthew et al. (2023)(London) [21]	*N* = 22 primary care teams; 159 participants; 15 teams in focus groups; 21 teams with reflection survey data	Reflection survey data; open-ended survey data; focus group transcripts; thematic analysis	CAPACITI: a 10-module virtual education program (1 h/month) teaching primary care teams to provide early palliative care; included interactive webinars, cheat sheets, 30-day tasks, mentorship with a palliative care specialist	Changes in practice derived from CAPACITI; usefulness of specific program elements; barriers and challenges to implementation; confidence and competence in applying the palliative approach	Participants reported that the multifaceted approach of CAPACITI was useful in building confidence and competence; three main themes: changes in practice, utility of components, barriers to implementation; teams reported early identification of patients and initiation of conversations about serious illness as the main changes	Some concerns
Kwok et al., 2022 (Caribbean) [22]	Healthcare professionals participating in the Project ECHO program in Caribbean countries, including doctors and other professionals involved in the care of patients with palliative needs	Program evaluation; participant feedback; analysis of session attendance; assessment of perceived changes in clinical practice	Project ECHO: a virtual educational model based on tele-mentoring, with regular case discussion sessions, interactive teaching, and support from palliative care specialists	Knowledge in palliative care; professional confidence; clinical management capacity; interprofessional collaboration	Improvement in the confidence and knowledge of participants in palliative care; strengthening of collaboration among professionals; increased capacity to manage patients with palliative needs in contexts with limited access to specialists; intervention considered viable and scalable	Some concerns
Forbat et al., 2024 (United Kingdom) [23]	Six specialized palliative care services involving multiple care homes in England and Scotland; healthcare professionals and institutionalized residents with palliative needs	Semi-structured interviews; workshops with stakeholders; meeting records (Needs Rounds); healthcare utilization data; quality of care indicators; assessment questionnaires (e.g., quality of dying and care)	Palliative Care Needs Rounds: regular meetings between specialized palliative care teams and professionals from long-term care institutions, focused on early patient identification, advance care planning, symptom management, and shared decision-making	Quality of end-of-life care; advance care planning; hospitalizations; place of death; team training and confidence; integration between services	The intervention increased the team’s confidence and competence in managing palliative care; improved communication and advance planning; strengthened integration between services; favored care in the patient’s preferred location and reduced potentially avoidable hospitalizations; considered viable and applicable on a large scale	High risk of bias
Reymond et al., 2005 (Australia) [24]	Primary healthcare professionals in rural areas (including general practitioners and other professionals involved in care)	Evaluation of the program through participant feedback; analysis of changes in clinical practice; records of participation and implementation of the strategy	Educational and support strategy to increase the capacity for palliative care in rural primary care, including training, specialist support, and skill development for managing patients with advanced diseases	Professional training; confidence in providing palliative care; integration with specialized services; changes in clinical practice	Improvement in the confidence and competence of primary healthcare professionals in managing palliative care; increased capacity to provide care locally; strengthening of integration with specialized services; intervention considered useful for rural contexts with access to specialists	Some concerns
Kavalieratos et al., 2022 (USA) [25]	*N* = 30 adults with NYHA class III or IV heart failure; 20 intervention; 10 control; 5 deaths during the study	Kansas City Cardiomyopathy Questionnaire (KCCQ); Edmonton Symptom Assessment Scale-Revised (ESAS-r); Medical Outcomes Study Social Support Survey; Patient Health Questionnaire-8; Hospital Anxiety and Depression Scale; Functional Assessment of Chronic Illness Therapy Spiritual Well-Being Scale; Patient Dignity Inventory; Advance Care Planning Engagement Survey; semi-structured interviews	PENPal-HF: training of outpatient cardiology nurses in palliative care principles (symptom burden, patient priorities, QoL, advance care planning) applied in quarterly visits over 56 weeks	Feasibility; acceptability; patient satisfaction; symptom burden; quality of life; advance care planning; perception of improvement	70% completed the study by week 56; 93.75% satisfied with the care; 87.5% agreed that everyone with heart failure should receive the intervention; 93.75% reported improvement in physical symptoms, mood, and/or quality of life	Some concerns
Brown et al., 2018 (Canada) [26]	*N* = 361,951 deceased adults; 11,006 physicians; 95.3% general practitioners; 4.7% specialists; 74.2% family doctors	ICES administrative data (OHIP, RPDB, Physician Database);algorithm for specialist identification. Delivery models: (1) low engagement (53.0%); (2) exclusive generalist (21.2%); (3) consultancy (14.7%); (4) exclusive specialist (11.1%)	Service models: (1) low engagement (53.0%); (2) exclusive generalist (21.2%); (3) consultancy (14.7%); (4) exclusive specialist (11.1%)	Access to palliative care; generalist-specialist coordination; urban-rural distribution	53.0% of the deceased without medical palliative care; 95.3% of care by generalists; only 2.7% by specialists in rural areas; family doctors responsible for >99% of rural care	Some concerns
Nguyen et al., 2018 (USA) [27]	Patients with non-small cell lung cancer (stages II–IV) and their family caregivers; recruited from three community centers of the Kaiser Permanente system	Functional Assessment of Cancer Therapy–Lung (FACT-L); Functional Assessment of Chronic Illness Therapy–Spirituality; City of Hope Quality of Life Tool; measures of psychological distress; assessment of healthcare service utilization	Nurse-led palliative care intervention, adapted for community practice, including symptom management, psychosocial support, patient/caregiver education, and structured follow-up; compared to usual care (quasi-experimental two-phase design)	Quality of life (physical, emotional, functional, and spiritual domains); suffering; use of health services; caregiver preparation and burden	Significant improvement in the physical, emotional, and functional domains of quality of life in patients from the intervention group (*p* < 0.01), maintained for up to 3 months; caregivers showed improvement in physical, spiritual well-being, and preparedness for care; no significant difference in the use of services or psychological distress; intervention considered effective and applicable in a community context	Some concerns

Note: ICES = Institute for Clinical Evaluative Sciences; OHIP = Ontario Health Insurance Plan; RPDB = Registered Person’s Database.

## Data Availability

No new data were created or analyzed in this study. Data sharing is not applicable to this article.

## Supplementary Materials

The following supporting information can be downloaded at: https://www.mdpi.com/article/10.3390/medsci14060690/s1, PRISMA 2020 Checklist.
